# The relationship between obesity and elderly suicide rates: a cross-national study

**DOI:** 10.5249/jivr.v2i2.63

**Published:** 2010-06

**Authors:** Ajit Shah

**Affiliations:** ^a^University of Central Lancashire, Preston, United Kingdom and Consultant Psychiatrist, West London Mental Health NHS Trust, London, United Kingdom.

## Abstract

**Background:**

An inverse relationship between obesity and suicide has been observed in younger adults, but this has not been examined in the elderly.

**Methods:**

A cross-national ecological study examined the independent relationship between the prevalence of obesity and elderly suicide rates, by controlling for potentially confounding variables, using data from the World Health Organization and the United Nations.

**Results:**

Elderly suicide rates in females were independently associated with the prevalence of obesity.

**Conclusions:**

Caution should be exercised in attributing a causal relationship from this cross-sectional ecological study due to ecological fallacy and requires confirmation in individual-level case-control or cohort studies.

## Introduction

An inverse relationship between body mass index (BMI) and suicide has been observed in male Swedish conscripts,^1^ male American healthcare professionals,^2^ and Americans^3^ and Norwegians ^4^ in the general population. This relationship was sustained after controlling for several confounding variables including smoking, alcohol intake, education, employment, marital status, social support, physical activity, medical illness, depression, antidepressant use.^2,4^

Collectively these studies, focusing on younger age groups, suggested an inverse relationship between obesity and suicide in both sexes. This relationship has been poorly examined in the elderly, although this inverse relationship has been observed in both those under and over the age of 50 years,^4^ but another study reported that this relationship was weaker in older men.^2^ Therefore, a cross-national ecological study with the null hypothesis that there will be no relationship between the prevalence of obesity and national elderly suicide rates was conducted.

## Methods

Data on elderly suicide rates for both sexes in the age-bands 65-74 years and 75+ years was ascertained from the World Health Organization (WHO) (http://www.who.int/whosis/ database/mort/table1.cfm). The median (range) year for the data on suicide rates was 2001 (1991-2002). Data on the national prevalence rate of obesity was also ascertained from the WHO (http://www.who.int/whosis/database/core/core select.cfm). The WHO had ascertained this information from national household surveys and defined obesity as BMI≥30.0 Kg/m^2^. The median (range) year for the data on the national prevalence rate of obesity was 2003 (2000-2006). The univariate relationship between elderly suicide rates in both sexes in both the age-bands and the prevalence of obesity was examined using Spearman’s rank correlation.

Elderly suicide rates are associated with socio-economic status, income inequality, life expectancy, educational attainment and marital status.^2,4^ Also, studies examining the relationship between obesity and the risk of suicide risk in younger adults controlled for several confounding factors including education, marital status, social support and socio-economic status.^2,4^ Therefore, the independent association between obesity and elderly suicide rates was examined using multiple regression analysis with the Enter method. Suicide rate was the dependent variable. Independent variables included in the analysis were the national prevalence rate of obesity, socioeconomic status measured by the gross national domestic product (GDP), income in equality measured by the Gini coefficient, life expectancy, educational attainment measured by the Education Index, rates of marriage and divorce rates. Data on the GDP and life expectancy were also ascertained from the WHO (http://www.who.int/countries/en/)  for the year 2002. Data on the Gini coefficient and the Education Index were ascertained from the United Nations Development Programme (http://hdr.undp.org/en/media/HDR_20072008_Tables.pdf) . The median (range) year for the data on the Gini coefficient was 2000 (1990-2003). Data on the rates of marriage and divorce rates was ascertained from the United Nations Demography Yearbook http://unstats.un.org/unsd/demographic /products/dyb/dybsets/2006%20DYB.pdf x for the year 2002.

## Results

Full data set of the national prevalence rate of obesity and suicide rates were available in males and females for 35 and 40 countries respectively. The prevalence of obesity and the suicide rates for each country for females and males are illustrated inTables 1  and 2 respectively. There were significant negative correlations between the prevalence of obesity in females and the suicide rates in females aged 65-74 (rho=-0.42, P=0.008) and 75+ (rho=-0.36, P=0.022) years; this was not observed in males in both the elderly age-bands. There was a highly significant positive correlation between male and female suicide rates in both the 65-74 years (rho=+0.87, P<0.00001) and 75+ years (rho=+0.89, P<0.00001) age-bands.

**Table:** Table 1: **Suicide rates in elderly females (per 100,000 of the relevant age-band) and female obesity**

Country	Prevalence of obesity (%)	Female suicide rate 65-74 years	Female suicide rate 75+ years
Armenia	15.5	1.6	1.7
Belgium	13.4	13.6	15.6
Bosnia	25.2	6.5	11.1
Brazil	13.1	2.6	2.6
Canada	13.9	4.9	2.8
Chile	25	1.5	2.2
China	3.4	39.2	61.2
Columbia	16.6	0.7	0.7
Croatia	22.7	19.4	31.3
Czech Republic	16.3	8.7	17
Denmark	9.1	12.6	10.9
Egypt	46.6	0	0
Estonia	14.9	11	23.1
Finland	13.5	11.1	7.5
Germany	12.3	10.8	18.2
Greece	18.2	0.8	2.2
Hungary	18.2	15.1	34.6
Ireland	12	0.8	1.7
Italy	8.9	5.7	5.9
Japan	3.3	19.6	26.1
Latvia	19.5	13.3	24.6
Lithuania	19.2	26.9	28.2
Mexico	28.1	1.1	1.2
New Zealand	23.2	1.5	3.2
Norway	5.9	4.6	3.2
Poland	19.9	7.8	7.6
Moldavia	18.2	10.6	14.4
Romania	9.5	7.7	8.8
Seychelles	35.2	0	0
Singapore	7.3	13	23.7
Slovakia	15	7.5	9.6
Slovenia	13.8	14.4	24
Spain	13.5	6.7	8.5
Sweden	9.5	9.4	12.7
Switzerland	7.5	17.9	23.9
Turkmenistan	10.3	4.9	17.5
Ukraine	11.3	13.5	18.7
United Kingdom	23	3.4	3.7
USA	33.2	4	4
Uzbekistan	7.1	5.6	8.7

**Table:** Table 2: **Suicide rates in elderly males (per 100,000 of the relevant age-band) and male obesity**

Country	Prevalence of obesity (%)	Male suicide rate 65-74 years	Male suicide rate 75+ years
Belgium	11.9	36.5	86.8
Bosnia	16.5	12.9	32.7
Brazil	8.9	13	17.9
Canada	15.9	16.9	22.7
Chile	19	31.3	31.9
China	2.4	43.7	84.2
Columbia	8.8	17	17.4
Croatia	21.6	67.5	108
Czech Republic	13.7	34	71.2
Denmark	9.8	34	46.6
Estonia	13.7	62.9	81.5
Finland	14.9	39.9	50.3
Germany	13.6	29	60.9
Greece	26	8.3	9.3
Hungary	17.1	73.9	121.10
Ireland	14	19.1	12.7
Italy	7.4	17.9	32.4
**Japan**	2.9	42.7	42.7
Latvia	11.9	45.2	70.2
Lithuania	20.6	78.4	84.9
Mexico	18.6	9.7	20.7
New Zealand	21.9	20.7	20.7
Norway	6.4	23.7	30
Poland	15.7	34.3	28.7
Romania	7.7	33	35.1
Seychelles	15	0	0
Singapore	6.4	21	51.1
Slovakia	13.5	38.5	42.6
Slovenia	16.5	75.1	106.7
Spain	13	20.9	41
Sweden	10.4	29.5	42.2
Switzerland	7.9	43.5	81.3
United Kingdom	22.3	8.7	10.4
USA	31.1	22.7	42.4
Uzbekistan	5.4	19.8	11.5

 The characteristics of multiple regression analyses for females in both the elderly age-bands are illustrated in Table 3. On multiple regression analysis: the independent predictors of suicide rate in females aged 65-74 years were the national prevalence rate of obesity in females (P=0.001), divorce rates (P=0.013) and GDP (P=0.039); and the independent predictors of suicide rate in females aged 75+ years were the national prevalence rate of obesity in females (P=0.003) and divorce rates (P=0.026), and the independent association GDP approached significance (P=0.063).

**Table:** Table 3: **Multiple regression analysis for females in both elderly age-bands**

Variable	B	Unstandardized coefficient Standard Error	Standardized coefficient Beta	t	P value
Females 65-74 years:					
Constant	-27.6	77.4		-0.36	NS
Prevalence of obesity	-0.81	0.22	-0.62	-3.6	0.001
Marriage rate	-1.54	1.84	-0.15	-0.83	NS
Divorce rate	3.81	1.57	0.45	2.43	0.023
Education Index	-56.7	52.8	-0.29	-1.1	NS
Life expectancy	1.37	0.75	0.63	1.8	NS
GDP	0	0	-0.84	-2.2	0.039
Gini coefficient	0.23	0.19	0.22	1.2	NS
Females 75+ years:					
Constant	19.5	122		0.16	NS
Prevalence of obesity	-1.18	0.35	-0.57	-3.35	0.003
Marriage rate	-4.69	2.9	-0.28	-1.62	NS
Divorce rate	5.88	2.48	0.44	2.37	0.026
Education Index	-125.1	83.3	-0.4	-1.0	NS
Life expectancy	1.86	1.18	0.54	1.58	NS
GDP	0	0	-0.75	-1.95	0.063
Gini coefficient	0.41	0.3	0.25	1.35	NS

## Discussion

 The absence of a significant correlation between obesity and elderly suicide rates in males was not consistent with previous studies of younger adult males,^1-4^ although one study suggested that this relationship was weaker in older men.^2^ Also, BMI may be a less accurate measure of obesity in older people because it may be disproportionately influenced by physical illness and changes in muscle mass associated with ageing.^2^

The observed significant and independent negative correlation between obesity and elderly suicide rates in females rejected the null hypothesis and was consistent with observations from several studies of females.^3-4^ Caution should be exercised in attributing causation and the direction of causality from a cross-sectional ecological study due to ecological fallacy. Nevertheless, potential explanations for the observed inverse relationship require exploration.

The reported inverse relationship between the BMI and depression in older people^2^ may also explain the inverse relationship between obesity and the risk of suicide. However, another recent study simultaneously and paradoxically demonstrated an inverse relationship between obesity and suicide and a positive relationship between obesity and depression, and the authors' suggested that the relationship between obesity and suicide may be independent of depression.^4^

Suicidal behaviors are associated with low levels of serotonin in the brain. Several mechanisms may operate in the elderly to reduce levels of serotonin in the brain. First, higher BMI is associated with higher insulin levels,^5^ which may result in greater release of serotonin in the brain, and this may explain improvement in subjective well-being and depressive symptoms administration of insulin in older adults with type 2 diabetes.^6^ Second, higher BMI is also associated with higher levels of serum cholesterol and triglycerides, which may lead to an increase in brain serotonin levels.^7^ Third, estrogen improves depression in perimenopausal women^8^ because it alters serotonergic neurotransmitter and receptor function,^9^ and this may explain the inverse association between obesity and depression observed in post-menopausal women.^10^ Thus, the impact of obesity on insulin levels, cholesterol levels and estrogen levels may influence central serotoninergic function and lead to reduction in the risk of suicide in elderly women; this may be mediated both through improvement in depression and/or independent of depression.

Caution should be exercised in attributing a causal relationship and the direction of causality from this cross-sectional ecological study due to ecological fallacy. Nevertheless, the observed independent inverse relationship between obesity and elderly suicide rates in females suggests that there is a need to confirm this relationship in the elderly in individual-level case-control or cohort studies.
